# Effects of indoor cycling (spinning) exercise on body image and anthropometric measurements in women: An eight-week intervention study

**DOI:** 10.1371/journal.pone.0358959

**Published:** 2026-10-05

**Authors:** Rustam Akhmedov, Murat Kasap, Dzhiparkul Abdyrakhmanova, Gökhan Recep Aydın, Nilufar Khaidarova, Saltanat Kaiky, Ayça Genç, Bilal Demirhan

**Affiliations:** 1 Department of Coach Education, Kyrgyz-Turkish Manas University Faculty of Sports Sciences, Bishkek, Kyrgyzstan; 2 Department of Coach Education, Bartın University, Faculty of Sports Sciences, Bartın, Türkiye; Polytechnic University of Guarda, PORTUGAL

## Abstract

This pre-test post-test intervention study included forty women (n = 40) with a mean age of 31.75 ± 5.38 years. Participants performed 45-minute indoor group cycling (spinning) sessions three times per week for eight weeks. Body image perception, assessed using the Body Image Scale developed by Secord and Jourard [1] and adapted into Turkish by Hovardaoglu [2], and anthropometric measurements were evaluated before and after the intervention. Following the exercise program, total body image scores increased significantly from 137.30 ± 27.19 to 159.00 ± 20.81 (p < .001, r = 0.85). Significant reductions were also observed in body weight (from 71.35 ± 9.05 kg to 67.53 ± 8.03 kg, −3.82 kg), body mass index (from 27.05 ± 3.18 kg/m^2^ to 25.61 ± 2.89 kg/m^2^), and chest, waist, hip, and thigh circumference measurements (all p < .001). No statistically significant change was found in shoulder circumference (p > .05). These findings indicate that an eight-week indoor group cycling (spinning) exercise program positively influences both body image perception and selected anthropometric indicators in adult women. Indoor cycling may therefore be considered an effective group-based exercise approach supporting both physical and psychological well-being. However, as this was a pre-test post-test study without a control group, causal interpretations should be made with caution.

## 1. Introduction

Body image is a multifaceted concept encompassing an individual’s perceptions, thoughts, feelings, and evaluations regarding their own body. This concept is not limited to physical appearance alone, but also includes an individual’s perceptions of their body’s functionality, health, strength, and overall physical fitness [1,2]. Frederick and colleagues [3] emphasize that body image is not merely a product of individual experiences, but a dynamic construct shaped by social norms, cultural values, gender roles, and media influences. In this context, body image, especially in women, takes on a more complex structure, influenced by age related physical changes, societal expectations, and aesthetic concerns [4]. Body image is closely related to an individual’s psychological well-being and plays a determining role in many psychosocial variables such as self-esteem, mental health, social relationships, and life satisfaction [5]. Negative body image perception is associated with low self-esteem, anxiety, depressive symptoms, and unhealthy behavioral patterns, while positive body image perception is considered an element that supports both the physical and mental health of the individual. It has been reported that body dissatisfaction is a common problem in adult populations, and this condition can persist throughout life, especially in women [6]. Therefore, interventions aimed at improving body image constitute an important area of research from a public health and sports science perspective.

“Many studies have shown that regular physical activity is an effective tool for improving individuals’ perceptions of body image [7–10]. This effect of exercise is not limited to physical changes; it is also associated with the development of an individual’s perception of functionality, strength, endurance, and mobility in relation to their body. A recent meta-analysis by Zhang et al. [9] demonstrated that physical exercise significantly improves body esteem in females, with aerobic exercise yielding larger effect sizes (g = 0.63) compared to other exercise modalities, whereas combined programs showed a smaller effect (g = 0.38). Similarly, Shu et al. [10] reported that aerobic exercise interventions effectively enhance body self-esteem, particularly among young adults, reinforcing the potential of structured aerobic programs such as spinning to positively influence body image perceptions. It has been reported that the relationship between physical activity level and depressive symptoms and psychological wellbeing is partially mediated by individuals’ body perceptions; and that the psychological benefits of exercise are more pronounced in individuals with a positive body image [11]. The functionality based body image model developed by Alleva et al. [12] shows that individuals are beginning to evaluate their bodies not only esthetically, but also based on how effectively and healthily they function. This approach provides an important theoretical framework for explaining the positive effects of exercise on body image.”

In recent years, group based and music accompanied exercise types have gained increasing attention due to their potential effects on both physical fitness and psychological well-being. Indoor cycling exercises (spinning) are a structured form of aerobic exercise performed on stationary bikes, combining elements of rhythm, music, and group motivation. Spinning exercises are widely preferred because they increase cardiorespiratory endurance, strengthen lower extremity muscles, and provide high energy expenditure [13,14].

Systematic review studies examining the effects of indoor cycling (spinning) exercises on cardiorespiratory fitness, metabolic health, and overall physical well-being have shown that this type of exercise is a safe and effective aerobic intervention for different age and population groups [15]. Notably, most studies on indoor cycling have focused on women with overweight or obesity [16], which is particularly relevant given that the present study’s sample (mean BMI 27.05 kg/m^2^) falls within the overweight range. There are studies in the literature examining the effects of indoor cycling exercises on body composition, cardiovascular fitness, and metabolic parameters [16,17]. However, the majority of studies on spinning exercises in women focus on physical outcomes; psychological variables such as body image are mostly addressed secondarily or indirectly.

It has been reported that perceived body weight in young and adult individuals in Turkey shows significant relationships with levels of depression, anxiety, and stress [18]. It appears that there are limited intervention based studies that simultaneously examine the effects of a structured spinning intervention on both body image and anthropometric measurements, particularly in adult women aged 30 and over. This situation highlights the need for research that holistically evaluates the physical and psychological effects of exercise.

In this context, the aim of the current study is to examine the effects of an eight week indoor group cycling (spinning) exercise program on adult women’s body image perceptions and anthropometric measurements. The study aims to provide a scientific contribution to the planning of exercise programs for women by revealing the role of exercise not only on physical but also on psychological outcomes.

## 2. Materials and methods

### Participants

A total of forty healthy women (n = 40) with an average age of 31.75 ± 5.38 years voluntarily participated in this study. It was stated that the participants had no chronic diseases, no musculoskeletal disorders, and had not participated in a regular exercise program in the last six months before being included in the study. Before the study, all participants were provided with detailed information about the study’s purpose, the exercise program to be implemented, potential risks, and data privacy; written informed consent forms were obtained from individuals who agreed to participate in the study.

Although an a priori sample size calculation was not conducted before participant recruitment, a post-hoc power analysis was performed to evaluate the statistical power of the achieved sample size. Using G*Power (version 3.1.9.7) with the observed effect sizes from the present study, α = 0.05, and two-tailed tests, the achieved power exceeded 0.95 for the primary outcome variables (body image total score and body weight). For shoulder circumference, the achieved power was moderate (≈0.52), consistent with the small observed effect size. It is important to note that the sample size (n = 40) was determined based on practical considerations and the exploratory nature of this investigation. The absence of a priori sample size calculation was primarily due to the lack of previous studies with comparable effect sizes in this specific population and intervention context, which made it difficult to estimate an expected effect size a priori. Furthermore, the exploratory nature of this study, which aimed to generate effect size estimates for future research, justified a flexible approach to sample size determination. While a priori power calculation is ideal, the achieved sample size in this study provided sufficient power to detect large effects, which is consistent with effect sizes reported in previous exercise intervention studies on body image (Cohen’s d = 0.80; Hausenblas & Fallon, 2006) [19].

Participants were included in the study only if they completed both the pre-test and post-test assessments, and all analyses were performed using the final complete dataset of 40 participants.

The research was conducted in accordance with the principles of the Declaration of Helsinki, and the study protocol was approved by the Ethics Committee of the Kyrgyz State Academy of Sports (Ethics Committee Approval No: 2025-168/4).

**Inclusion criteria for the study**:

- Be 18 years of age or older,
- Have no chronic illnesses or physical disabilities,
- Have not engaged in regular exercise within the past six months,
- Agree to participate in the study and complete the eight week intervention.

### Study design

This study employed a single group pre-post intervention design to examine the effects of an eight-week indoor cycling (spinning) exercise program on body image perception and anthropometric characteristics. Although a control group was not included, this study was designed as an initial investigation to evaluate the magnitude of changes following a structured spinning intervention. The absence of a control group was primarily due to practical constraints in participant recruitment and adherence, as well as the exploratory nature of this investigation aimed at generating effect size estimates. Moreover, the primary objective of this preliminary study was to assess within-subject changes and to provide preliminary evidence that could inform the design of future randomized controlled trials, rather than to establish causal inference. Similar pre-post designs have been used in preliminary exercise intervention studies to inform the design of future randomized controlled trials [10]. Measurements were collected at two time points: before the intervention (pre-test) and after the intervention (post-test). Data collection and testing procedures were conducted between July 15, 2025 and September 15, 2025.

### Exercise intervention

The exercise intervention consisted of indoor group cycling (spinning) exercises performed three times a week for eight weeks. Each exercise session lasted a total of 45 minutes and was conducted under the guidance of an experienced instructor. The general structure of the sessions is as follows:

- Warm-up (5–7 min): Low intensity pedaling and dynamic movements to increase joint range of motion.
- Main exercise section (30–35 minutes): Structured spinning exercises with music, alternating between moderate and moderate high intensity, steady pace riding, sprints, and climbing simulations.
- Cool-down (5–7 min): Low intensity pedaling, light stretching, and breathing exercises.

Exercise intensity was monitored using the Borg Rate of Perceived Exertion Scale (6–20) [20]. The aim was for participants’ perceived exertion levels during exercise to remain within the range of 13–15, which was considered to correspond to approximately 70–85% of their maximum heart rate. This is supported by previous meta analytic evidence, which showed strong relationships between the Borg Rate of Perceived Exertion Scale and physiological indicators such as heart rate and oxygen consumption, and that it is a valid tool for monitoring exercise intensity [21]. This effort score range of 13–15 generally represents an effort level described as “somewhat difficult to difficult,” corresponding to moderate-to-high intensity aerobic exertion. Additionally, the in session workload level was monitored through speech tests and participant feedback. A detailed step-by-step protocol of the indoor cycling exercise intervention is available on protocols.io. To ensure reviewer anonymity, a blinded link is provided here: https://www.protocols.io/blind/B1D0AE55878411F199810A58A9FEAC02.

### Data collection tools

#### Body image scale.

The Body Image Scale, developed by Secord and Jourard [22] to assess participants’ perceptions of body image and adapted into Turkish by Hovardaoglu [23], was used. The scale is a one dimensional measurement tool consisting of 40 items in total, using a 5 point Likert scale, which measures individuals’ satisfaction levels with different parts of their bodies. High scores on the scale indicate a more positive body image. In the Turkish adaptation study, the internal consistency coefficient was reported as α = 0.95. The scale was administered twice, before and after the exercise program.

#### Anthropometric measurements.

As part of the anthropometric assessments, the participants’ body weight (kg), height (cm), body mass index (kg/m^2^), shoulder circumference (cm), chest circumference (cm), waist circumference (cm), hip circumference (cm), and thigh circumference (cm) were measured. All measurements were taken by the same trained researcher under standardized conditions, following standard anthropometric measurement procedures. To enhance measurement reliability, each circumference measurement was taken twice, and the average of the two measurements was recorded. If the difference between the two measurements exceeded 0.5 cm, a third measurement was taken. The intra-observer technical error of measurement (TEM) was calculated for each variable, with coefficients of variation ranging from 0.5% to 1.2%, indicating acceptable reliability. Body mass index was calculated by dividing body weight by the square of height in meters.

### Statistical analysis

All statistical analyses were performed using the complete dataset consisting of 40 participants and were conducted using jamovi statistical software (version 2.x; The jamovi Project). Descriptive statistics were calculated for all variables and are presented as mean ± standard deviation. Prior to inferential analyses, the normality of change scores (post-test minus pre-test values) was assessed using the Shapiro-Wilk test. Based on distributional assumptions, paired samples t-tests were applied for normally distributed variables, whereas the Wilcoxon signed rank test was used for variables that did not meet normality assumptions. Effect sizes were calculated and reported as Cohen’s d for parametric comparisons and as r for non-parametric comparisons, providing an estimate of the magnitude of observed effects. Statistical significance was set at p < 0.05 for all analyses.

Post-hoc power analyses (α = 0.05, two-tailed, n = 40) were conducted based on the observed effect sizes. The results indicated high statistical power for the primary outcome variables, supporting the study’s ability to detect meaningful changes following the intervention. For shoulder circumference, the achieved power was moderate (≈0.52), which was consistent with the small observed effect size. It should be noted that post-hoc power analyses are inherently limited and should be interpreted with caution.

Spearman correlation analyses were conducted to examine the relationships between changes in body image scores and changes in anthropometric measurements. Correlation coefficients (ρ) and corresponding p values were reported to assess the strength and significance of these associations.

## 3. Results

This section presents the findings regarding the effects of an eight week indoor cycling (spinning) exercise program on participants’ anthropometric measurements and body image perceptions.

### Demographic characteristics of participants

The demographic characteristics of the individuals participating in the study are presented in Table 1. The average age of the participants was 31.75 ± 5.38 years, and the average body mass index was determined to be 27.05 ± 3.18 kg/m^2^. These values indicate that the participants generally fall into the overweight category.

**Table 1:** Demographic characteristics of the participants (n = 40).

Variable	Mean ± SD	Min	Max
Age (years)	31.75 ± 5.38	23	43
Height (cm)	162.40 ± 6.03	147	174
Body Weight (kg)	71.35 ± 9.05	48	86
Body Mass Index (kg/m^2^)	27.05 ± 3.18	19.47	34.58

Note: Values are presented as mean ± standard deviation (SD). Body mass index (BMI) was calculated as body weight (kg) divided by height squared (m^2^).

### Findings related to anthropometric measurements

According to the results presented in Table 2, participants’ body weight decreased significantly after the intervention period (p < .001), with a very large effect size (r = 0.87). Similarly, body mass index values showed a statistically significant reduction following the intervention (p < .001), accompanied by a very large effect size (d = 1.91).

**Table 2:** Effects of the 8-week indoor cycling (spinning) exercise program on anthropometric measurements (n = 40).

Variable	Pre-test (Mean ± SD)	Post-test (Mean ± SD)	Test	p value	Effect Size
Body Weight (kg)	71.35 ± 9.05	67.53 ± 8.03	Wilcoxon	<.001*	r = 0.87
Body Mass Index (kg/m²)	27.05 ± 3.18	25.61 ± 2.89	Paired t-test	<.001*	d = 1.91
Shoulder Circumference (cm)	107.78 ± 8.92	107.90 ± 9.34	Wilcoxon	.326	r = 0.16
Chest Circumference (cm)	98.55 ± 8.54	96.95 ± 8.12	Wilcoxon	<.001*	r = 0.66
Waist Circumference (cm)	88.12 ± 11.63	83.62 ± 9.88	Wilcoxon	<.001*	r = 0.78
Hip Circumference (cm)	104.47 ± 7.11	102.05 ± 5.82	Wilcoxon	<.001*	r = 0.70
Thigh Circumference (cm)	56.67 ± 6.14	55.38 ± 5.41	Wilcoxon	<.001*	r = 0.70

Note: Values are presented as mean ± SD. The Wilcoxon signed-rank test was used for body weight and circumference measurements due to non-normal distribution of change scores, whereas the paired-samples t-test was applied for body mass index. Effect sizes are reported as r for non-parametric comparisons and as Cohen’s d for parametric comparisons. Statistical significance was set at p < 0.05.

Circumference measurements demonstrated significant reductions in chest, waist, hip, and thigh regions after the eight-week indoor cycling program (all p < .001), with large effect sizes (r ranging from 0.66 to 0.78), indicating meaningful anthropometric changes.

In contrast, shoulder circumference did not show a statistically significant change between pre-test and post-test measurements (p > .05), and the observed effect size was small (r = 0.16).

### Findings regarding the body image scale

The results of the participants’ pre-test and post-test scores obtained from the Body Image Scale are presented in Table 3. Following the eight-week indoor cycling (spinning) exercise program, a statistically significant increase was observed in total body image scores (p < .001). According to the Wilcoxon signed-rank test results, this improvement was accompanied by a very large effect size (r = 0.85), indicating a substantial enhancement in body image perception after the intervention.

**Table 3:** Changes in body image scale scores following the 8-week spinning program (n = 40).

Variable	Pre-test (Mean ± SD)	Post-test (Mean ± SD)	Mean Change (Δ)	Test	p value	Effect Size
Body Image Total Score	137.30 ± 27.19	159.00 ± 20.81	+21.70 ± 14.32	Wilcoxon	<.001*	r = 0.85

Note: The Body Image Scale consists of 40 items, with a maximum possible score of 200. Higher total scores indicate a more positive body image perception. Values are presented as mean ± SD. The mean change of +21.70 points represents a 15.8% improvement from baseline. The Wilcoxon signed rank test was applied due to non-normal distribution of change scores. Statistical significance was set at p < 0.05. *p < .001.

### Correlation between body image and anthropometric changes

To examine the relationship between improvements in body image and changes in anthropometric measurements, Spearman correlation analyses were performed. As shown in Table 4, significant negative correlations were observed between changes in body image scores and changes in body weight (ρ = −0.42, p = 0.007), BMI (ρ = −0.38, p = 0.015), chest circumference (ρ = −0.35, p = 0.028), waist circumference (ρ = −0.51, p = 0.001), hip circumference (ρ = −0.41, p = 0.009), and thigh circumference (ρ = −0.33, p = 0.037). The strongest correlation was found between body image improvement and waist circumference reduction. No significant correlation was observed between body image change and shoulder circumference change (ρ = −0.08, p = 0.623) (Fig 1).

**Table 4:** Spearman correlation analysis between body image change scores and anthropometric change scores (n = 40).

Variable (Δ = Post-test – Pre-test)	Spearman ρ	p value
Δ Body Weight	−0.42	0.007**
Δ Body Mass Index	−0.38	0.015*
Δ Shoulder Circumference	−0.08	0.623
Δ Chest Circumference	−0.35	0.028*
Δ Waist Circumference	−0.51	0.001**
Δ Hip Circumference	−0.41	0.009**
Δ Thigh Circumference	−0.33	0.037*

Note: *p < .05, **p < .01. Body image change scores were calculated as post-test total score minus pre-test total score. Higher body image change scores indicate greater improvement in body image perception. Negative correlations indicate that greater reductions in anthropometric measurements are associated with greater improvements in body image.

**Fig 1:**
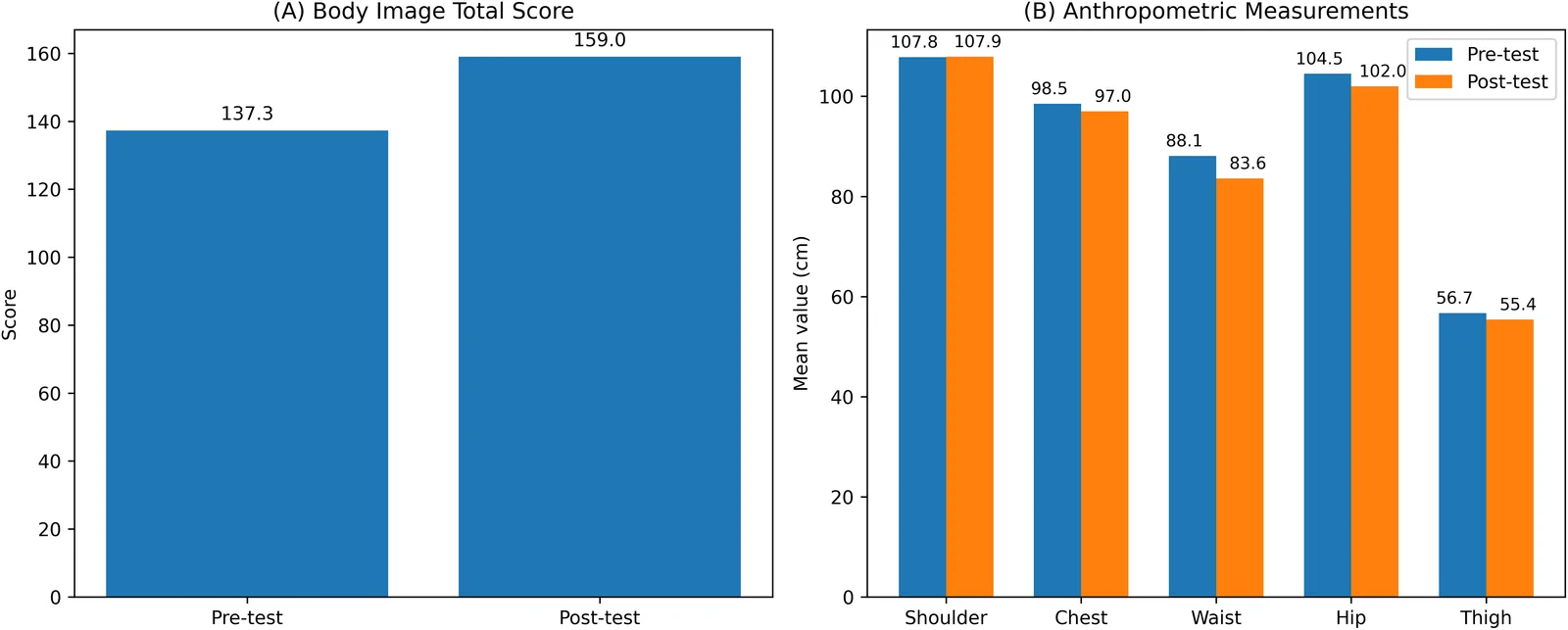
(A) Changes in body image total scores and (B) changes in anthropometric measurements before and after the 8 week indoor cycling (spinning) exercise program.

## 4. Discussion

The primary aim of this study was to investigate the effects of an eight week indoor group cycling (spinning) exercise program on adult women’s body image perceptions and anthropometric measurements. The findings indicate that spinning exercises have significant and positive effects on both psychological and physical outcomes.

The most powerful and striking finding of this study is the significant improvement observed in participants’ body image perceptions after an eight week spinning exercise program. It has been reported that physical activity based interventions have positive effects on individuals’ body image and weight related concerns, and that these effects are observed not only in adults but also in different age groups [24].

The statistically significant increase in the total Body Image Scale score from 137.30 to 159.00, accompanied by a very large effect size (r = 0.85), indicates that the observed improvement is not only statistically meaningful but also substantial in practical and psychological terms. This result reveals that exercise based interventions can be a powerful tool for improving body image in women and constitutes one of the main contributions of the present study. This finding also aligns with theoretical approaches that emphasize body image as a dynamic construct that can change throughout life through experiences, environmental factors, and behavioral interventions, rather than a static trait [25]. The mean improvement of +21.70 points (15.8% increase from baseline) further underscores the clinical and practical significance of the intervention.

Importantly, the magnitude of improvement in body image cannot be explained solely by anthropometric changes. Spinning exercises may contribute to the restructuring of body image through mechanisms such as increased cardiorespiratory endurance, enhanced perceptions of physical fitness, and improved awareness of bodily functionality. These functional gains may promote a more positive and appreciative perception of the body beyond changes in physical appearance. This interpretation is supported by the correlation analyses, which showed moderate associations between body image improvement and anthropometric changes, suggesting that other factors such as increased fitness, group dynamics, and psychological empowerment also contribute to the observed effects.

It has been suggested that positive body image is more strongly supported when motivation for exercise participation is health and functionality based rather than appearance focused [26]. The functionality based body image model described by Alleva et al. [12] emphasizes that individuals increasingly evaluate their bodies based on what they can do rather than how they look. In this context, it is plausible that spinning exercises positively transformed participants’ perceptions of their bodies. Indeed, previous studies have shown that the psychological benefits of physical activity are closely linked to body perception, and that this relationship is particularly pronounced in women [11].

In addition, performing spinning exercises in a group setting, accompanied by music and under the guidance of an instructor, may represent an important psychosocial mechanism supporting positive changes in body image. It has been shown that the environment in which exercise is performed (e.g., indoor versus outdoor settings) can influence body image outcomes, and that structured indoor activities such as group cycling are associated with positive body image [27]. Group based exercise settings are known to enhance social interaction, motivation, and enjoyment, all of which have been reported to positively influence body image [13,14]. This interpretation is further supported by the findings of Tiggemann [4] and Frederick et al. [3], who emphasize that body image is shaped not only by individual factors but also by social context.

The findings of the present study are largely consistent with previous research demonstrating the positive effects of regular physical activity on body image. Aerobic exercise interventions have been reported to yield more pronounced improvements in body image compared to resistance based programs, particularly among women with pre-existing negative body perceptions [28]. Meta-analyses conducted by Hausenblas and Fallon [19] and Campbell and Hausenblas [8] have consistently shown that exercise based interventions significantly improve body image, especially in women. Similarly, Bassett-Gunter et al. [7] reported beneficial effects of regular physical activity on body image, self-esteem, and psychological well-being. These findings support the main psychological outcome of the current study and suggest that spinning exercises represent an effective exercise modality in this context.

When anthropometric measurements were examined, the significant reductions observed in body weight (−3.82 kg), body mass index (−1.44 kg/m^2^), and chest (−1.60 cm), waist (−4.50 cm), hip (−2.42 cm), and thigh (−1.29 cm) circumference measurements were consistent with existing literature. Previous studies have reported that participation in indoor fitness activities is associated with more positive body perception and favorable changes in body measurements [29]. Similarly, research has shown that spinning exercises can lead to reductions in anthropometric measures by increasing overall energy expenditure [16,17]. The pronounced reduction in waist circumference (−4.5 cm) observed in the present study may be considered clinically meaningful, given its association with abdominal adiposity and cardiometabolic risk. However, it is important to acknowledge that this change may be partially mediated by dietary factors, as nutritional habits have been shown to influence anthropometric responses to exercise interventions [30]. Since the present study did not monitor participants’ dietary intake, it is not possible to definitively attribute the observed changes solely to the spinning program. It remains unclear whether the reductions in body weight and circumferences resulted directly from increased energy expenditure through exercise, from spontaneous or conscious changes in dietary habits during the intervention period, or from a combination of both. Without dietary control or assessment, the independent contribution of the exercise intervention to the observed anthropometric improvements cannot be isolated. Future studies should incorporate dietary assessments and nutritional counseling to better understand the interplay between exercise and dietary factors in body composition changes.

In contrast, the absence of a statistically significant change in shoulder circumference (+0.12 cm, p > .05) suggests that exercise induced adaptations may occur in a region specific manner. Aerobic based spinning exercises primarily target energy expenditure and fat metabolism, particularly in central body regions, whereas changes in upper body circumferences may require longer intervention durations or different training stimuli. Cadore et al. [31] reported that trunk related adaptations tend to be more pronounced than peripheral changes during short term aerobic interventions, providing a physiological explanation for the present findings.

It is important to acknowledge that the relationship between exercise participation and psychological outcomes may vary across cultural contexts. A study conducted in Mexico by Gómez Chávez et al. [32] found that physical activity did not consistently moderate psychological distress in the general adult population, suggesting that cultural factors and regional characteristics may influence the mental health benefits derived from exercise. While the present study observed significant improvements in body image among Turkish women, the generalizability of these findings to culturally diverse populations warrants further investigation. Future research should examine whether similar effects are observed in different cultural settings, and whether cultural norms regarding body image and exercise participation moderate the effectiveness of spinning interventions.

A notable contribution of the present study to the literature is the examination of body image outcomes within an intervention based design alongside anthropometric changes. While many previous studies have employed cross sectional designs or focused solely on physical outcomes, this study directly demonstrates the effects of a structured group based exercise intervention on body image, a key psychological variable, in adult women aged 30 years and older. Furthermore, the correlation analyses provide novel insights into the relationship between physical and psychological changes, revealing that while anthropometric improvements are associated with enhanced body image, the strength of these associations suggests that other factors also contribute to the psychological benefits of exercise.

In summary, the findings of this study indicate that indoor group cycling (spinning) exercises positively influence both body image perception and selected anthropometric indicators in adult women. In this respect, spinning exercises may be considered a promising exercise approach that supports not only physical fitness but also psychological well-being. These findings suggest that spinning programs can be effectively implemented as interventions to enhance women’s psychological health in fitness centers and community based exercise settings.

### Limitations

Although the findings of this study indicate that an eight-week indoor cycling (spinning) exercise program has positive effects on body image and certain anthropometric measurements in adult women, they should be interpreted in light of several limitations.

One of the primary limitations of the study is the use of a single group pre-test, post-test experimental design and the absence of a control group. Without a control group, it is not possible to attribute the observed changes exclusively to the exercise intervention, and causal interpretations should therefore be made with caution. Similar pre-post designs have been employed in preliminary exercise intervention studies to generate effect size estimates and inform the design of future randomized controlled trials [10]. However, the lack of a control group remains a methodological limitation that should be addressed in future research, as emphasized by Shu et al. [10] and others in the field.

The relatively homogeneous sample composition also represents a limitation of the study. The participants consisted exclusively of adult women, which restricts the generalizability of the findings to male individuals, younger populations, or older age groups. Additionally, the sample size (n = 40), while adequately powered to detect large effects as demonstrated by post-hoc power analysis, may still limit the generalizability of the findings to broader populations given the specific demographic characteristics of the participants (e.g., adult women aged 23–43 years from a single cultural context). The homogeneous nature of the sample, rather than the sample size per se, is the primary factor affecting generalizability. Furthermore, an a priori sample size calculation was not performed prior to data collection. The absence of a priori sample size calculation was primarily due to the lack of previous studies with comparable effect sizes in this specific population and intervention context, which made it difficult to estimate an expected effect size a priori. Furthermore, the exploratory nature of this study, which aimed to generate effect size estimates for future research, justified a flexible approach to sample size determination. While post-hoc power analysis indicated sufficient power for detecting large effects, the absence of a priori calculation may limit the study’s ability to detect smaller, yet potentially meaningful, effects. Future studies should conduct a priori power analyses based on the effect sizes reported in the present study to ensure adequate power for detecting smaller, yet potentially meaningful, effects.

Furthermore, dietary intake and eating habits were not monitored during the intervention period. Diet plays a significant role in anthropometric changes, and without its control, the independent contribution of the exercise intervention to the observed physical changes cannot be fully isolated. Participants may have consciously or unconsciously modified their dietary habits during the eight-week program, which could have contributed to the observed reductions in body weight and circumference measurements. Conversely, the absence of dietary monitoring also precludes the possibility of identifying whether any participants consumed a diet that could have attenuated the expected anthropometric improvements. This lack of dietary data represents a significant confounding factor that limits the interpretability of the anthropometric findings. Future studies should incorporate dietary assessments and nutritional counseling to better understand the interplay between exercise and dietary factors in body composition changes [30].

The findings are specific to a cohort of adult women in Turkey, and cross-cultural differences may influence the applicability of these results to other populations. As noted in previous research, the psychological benefits of physical activity can vary across cultural and regional contexts [32]. The generalizability of the present findings should therefore be confirmed through studies in diverse cultural settings.

With regard to anthropometric assessments, only body mass index and circumference measurements were used to evaluate physical changes. More detailed body composition parameters, such as body fat percentage, lean mass, and regional fat distribution, were not assessed using objective methods such as dual energy X-ray absorptiometry (DEXA) or bioelectrical impedance analysis (BIA). This limits a more comprehensive understanding of the physiological adaptations induced by the exercise intervention.

Exercise intensity was monitored using subjective methods, including the Borg Rating of Perceived Exertion Scale and the talk test. Objective physiological indicators such as heart rate monitoring, oxygen consumption, or metabolic thresholds were not employed. Consequently, the precise physiological load of the exercise sessions could not be quantified with high accuracy.

Finally, although the relationship between changes in body image and changes in anthropometric measurements was statistically examined in the present study (see Table 4), the correlational nature of this analysis does not establish causality. Future research employing structural equation modeling or mediation analyses may provide deeper insights into the interaction between psychological and physical adaptations resulting from exercise interventions.

## 5. Conclusion

This study demonstrated that an eight-week indoor group cycling (spinning) exercise program led to a statistically significant improvement in body image perception among adult women, along with favorable changes in several anthropometric measurements. The substantial increase observed in total body image scores (from 137.30 to 159.00), accompanied by a very large effect size (r = 0.85), highlights the strong psychological impact of the intervention.

The significant reductions observed in body weight (−3.82 kg), body mass index (−1.44 kg/m^2^), and chest (−1.60 cm), waist (−4.50 cm), hip (−2.42 cm), and thigh (−1.29 cm) circumference measurements indicate that indoor cycling exercises can positively influence body-related physical outcomes by increasing overall energy expenditure. In contrast, the absence of a significant change in shoulder circumference (+0.12 cm, p > .05) suggests that exercise-induced adaptations may occur in a region-specific manner, depending on the characteristics and duration of the exercise stimulus.

Beyond physical changes, the findings suggest that spinning exercises represent a promising exercise approach that appears to support psychological well-being in addition to physical fitness. The group-based structure of spinning sessions, combined with music and instructor guidance, may play an important role in fostering positive body perceptions by enhancing motivation, enjoyment, and social interaction. These psychosocial elements likely contribute to the observed improvements in body image beyond what can be explained by anthropometric changes alone, as supported by the moderate correlations observed between anthropometric changes and body image improvement.

Taken together, the results indicate that indoor group cycling appears to be a promising exercise model for improving body image and selected anthropometric indicators in adult women. For women who do not regularly engage in physical activity, spinning exercises may offer a sustainable and accessible alternative for initiating and maintaining an active lifestyle.

However, the pre-post design without a control group limits causal interpretations. Future randomized controlled trials with larger, more diverse samples and objective physiological monitoring are needed to confirm these preliminary findings and establish spinning as a comprehensive intervention approach. Additionally, the potential influence of dietary factors and cultural context on the observed outcomes should be systematically examined in future research.

## Supporting information

S1 File Data. (XLSX)
