# Supplementary material for: Effects of indoor cycling (spinning) exercise on body image and anthropometric measurements in women: An eight-week intervention study
Source: PLoS One. 2026 Oct 5;21(10):e0358959. doi: 10.1371/journal.pone.0358959 (PMC13637853; doi:10.1371/journal.pone.0358959)
Supplement: Supplementary file 1 [file pone.0358959.s003.docx]

**Dear Editor-in-Chief and Reviewers,**

We sincerely thank the Academic Editor and the reviewers for their valuable time and constructive feedback on our manuscript (PONE-D-26-08250). We have carefully considered all the points raised and have revised the manuscript accordingly. Below, we provide a point-by-point response to all comments. All changes in the revised manuscript are highlighted using the "Track Changes" feature.

**RESPONSES TO REVIEWER #1**

**Reviewer #1: Abstract**

**Comment:** The abstract clearly presents the main findings, but it omits essential information about the study design. In particular, it does not specify that this was a pre-post study without a control group, which affects the causal interpretation of the results (Shu et al., 2023, PLOS ONE, doi: 10.1371/journal.pone.0291045). Nor does it report the numerical values for the change in body image (137.30 to 159.00) or the absolute magnitude of the weight reduction (-3.82 kg). These data are presented in the tables and should be incorporated into the abstract to facilitate quantitative reading.

**Our Response:** We thank the reviewer for this valuable suggestion. We have revised the abstract to explicitly state that this was a pre-test post-test intervention study without a control group and have added a cautionary note regarding causal interpretations. We have also incorporated the numerical values for the body image scores and the absolute weight reduction as requested.

**Action Taken:** The abstract has been revised accordingly.

Line numbers in the revised manuscript: Abstract section.

**Reviewer #1: Introduction**

**Comment (Paragraph 2):** Physical exercise is presented as a tool that may be considered for improving body image, but no distinction is made between aerobic and strength-based modalities. The meta-analysis by Fan et al. (2024, BMC Public Health, doi: 10.1186/s12889-024-20861-7) showed that aerobic exercise yields an effect size of g=0.63 for body image in women, compared with g=0.38 in combined programmes. This specificity should be mentioned to justify the choice of spinning.

**Our Response:** We agree with the reviewer and have added the comparison of effect sizes between aerobic (g=0.63) and combined (g=0.38) programs from the Zhang et al. [29] (Fan et al.) meta-analysis. This distinction now clearly justifies our choice of a purely aerobic intervention like spinning.

**Action Taken:** The Introduction section has been updated.

Line numbers in the revised manuscript: Introduction, Paragraph 2.

**Comment (Paragraph 4):** The review by Chavarrias et al. (2019) is cited as general evidence, but it is omitted that most studies focused on indoor cycling have centred on women with obesity or overweight (Ratajczak et al., 2020, IJERPH, doi: 10.3390/ijerph17238718), which is relevant given that the sample in the present study (mean BMI 27.05 kg/m²) falls within the overweight range.

**Our Response:** This is an excellent point. We have now explicitly noted that most studies on indoor cycling focus on overweight or obese women and highlighted that our sample (mean BMI 27.05 kg/m²) falls within this range, which strengthens the relevance of our work.

**Action Taken:** The Introduction section has been updated.

Line numbers in the revised manuscript: Introduction, Paragraph 4.

**Reviewer #1: Materials and Methods**

**Comment (Participants):** No a priori sample size calculation is provided. Reporting post-hoc power does not replace an a priori calculation, and the authors themselves acknowledge its limitations. This should be clarified.

**Our Response:** We acknowledge this limitation and have clarified the reasoning further. We have expanded the explanation in the Participants section to justify the use of a post-hoc power analysis and the exploratory nature of the study.

**Action Taken:** The Participants section has been revised.

Line numbers in the revised manuscript: Materials and Methods, Participants.

**Comment (Study Design):** The pre-post design without a control group is the main methodological limitation and should be stated explicitly at this point, rather than only at the end of the manuscript.

**Our Response:** We agree and have now explicitly stated the limitation of the single-group pre-post design in the Study Design section. We also provided a strong justification for this choice, emphasizing the exploratory aim of generating effect size estimates for future research.

**Action Taken:** The Study Design section has been significantly revised.

Line numbers in the revised manuscript: Materials and Methods, Study Design.

**Comment (Anthropometric Measurements):** Only one measurement per variable is reported. It would be advisable to specify whether duplicate or triplicate measurements were taken, as well as the intra-observer coefficient of variation, given that body image may additionally modulate beliefs about body shape.

**Our Response:** We thank the reviewer for this suggestion. We have now clarified in the Anthropometric Measurements section that all circumference measurements were taken twice, with a third measurement taken if the difference exceeded 0.5 cm. We have also reported the intra-observer coefficients of variation, which ranged from 0.5% to 1.2%, indicating acceptable reliability.

**Action Taken:** The Anthropometric Measurements section has been expanded.

Line numbers in the revised manuscript: Materials and Methods, Anthropometric Measurements.

**Reviewer #1: Results**

**Comment (Section 3, Table 1):** There is an inconsistency: the text reports a mean BMI of 27.07 kg/m², whereas Table 1 gives 27.05 kg/m². This should be standardised.

**Our Response:** We thank the reviewer for catching this error. The value has been standardised to 27.05 kg/m² in both the text and Table 1.

**Action Taken:** The text has been corrected.

Line numbers in the revised manuscript: Results, Demographic Characteristics of Participants.

**Comment (Section 3, Table 2):** The change in shoulder circumference (107.78 to 107.90 cm) is virtually negligible and should be highlighted at the beginning of the section, rather than at the end, as an interpretative contrast that reflects the regional specificity of the aerobic stimulus.

**Our Response:** We agree with the reviewer's interpretation. We have restructured the paragraph in the Results section to present the significant changes in body weight and other circumferences first, and then placed the non-significant shoulder circumference change at the end, emphasizing it as a contrast that highlights the regional specificity of the aerobic stimulus.

**Action Taken:** The Results section has been reorganized.

Line numbers in the revised manuscript: Results, Findings Related to Anthropometric Measurements.

**Reviewer #1: Discussion**

**Comment (Paragraph 7):** Despite the findings, the interpretation of the psychological effects of spinning is reasonable; however, it should be noted that in Latin American populations exercise does not always act as a moderating factor for psychological distress (Gómez Chávez et al., 2022, RICCAFD, 11(2): 121-133). This cultural variability should be addressed when discussing the generalisability of the findings.

**Our Response:** This is an important point. We have incorporated this perspective into the Discussion section, acknowledging that the psychological benefits of exercise may vary across cultural contexts, as demonstrated by Gómez Chávez et al. (2022). We now discuss the need for future research in diverse cultural settings to confirm the generalizability of our findings.

**Action Taken:** The Discussion section has been updated.

Line numbers in the revised manuscript: Discussion, Paragraph 7.

**Comment (Paragraph 8):** The reduction in waist circumference (-4.5 cm) is described as "clinically significant", but this is not contextualised in relation to dietary habits, which may mediate this response (Meza Miranda, 2024, RICCAFD, 13(3): 274-285, doi: https://doi.org/10.24310/riccafd.13.3.2024.19808).

**Our Response:** We fully agree and have significantly expanded our discussion of this point. We now explicitly acknowledge that the observed waist circumference reduction may be partially mediated by dietary factors, which we did not monitor. This is now discussed as a significant confounding factor in both the Discussion and Limitations sections.

**Action Taken:** The Discussion and Limitations sections have been revised.

Line numbers in the revised manuscript: Discussion, Paragraph 8; Limitations.

**Reviewer #1: Conclusion**

**Comment:** Regarding the findings obtained, the conclusion is coherent, although, in my view, the assertion that spinning constitutes a "holistic approach" goes beyond what a design without a control group can support. The translational scope should therefore be qualified, and this term reserved for future studies using a randomised design, as the authors themselves acknowledge in lines 354--357.

**Our Response:** We thank the reviewer for this important clarification. We have removed the term "holistic" and replaced it with more cautious wording, such as "a promising exercise approach." We have also strengthened the concluding statements to emphasize that these findings are preliminary and require confirmation through future randomized controlled trials.

**Action Taken:** The Conclusion section has been revised.

Line numbers in the revised manuscript: Conclusion.

**RESPONSES TO REVIEWER #2**

**Reviewer #2: General Comment**

**Comment:** The manuscript is well developed, addresses an interesting topic, and has the potential to be published. However, several issues should be clarified and addressed before it can be considered for publication.

**Our Response:** We thank the reviewer for their positive and constructive feedback. We have carefully addressed each of the points raised, as detailed below.

**Comment (Control Group):** The authors should justify why a control group was not included, rather than simply stating that no control group was used and acknowledging this as a limitation.

**Our Response:** We have provided a detailed justification in the Study Design section. We now explain that the absence of a control group was due to practical constraints in participant recruitment and adherence, as well as the exploratory nature of this investigation aimed at generating effect size estimates. We also emphasize that the primary objective was to assess within-subject changes to inform future randomized controlled trials, not to establish causal inference.

**Action Taken:** The Study Design section has been significantly revised.

Line numbers in the revised manuscript: Materials and Methods, Study Design.

**Comment (Sample Size):** The authors should justify why an a priori sample size calculation was not performed. Although they acknowledge that the sample size is relatively small and that the findings cannot be generalized, this statement requires further justification. On what basis do the authors conclude that the sample is too small or not sufficiently representative? An a priori sample size calculation is essential to demonstrate that the study was adequately powered to detect the expected effects and to support the validity of the findings.

**Our Response:** We have significantly expanded our explanation in both the Participants and Limitations sections. The lack of an a priori calculation is justified by the absence of comparable effect size estimates in this specific population and intervention context at the time of study planning. The exploratory nature of the study justified a flexible approach to sample size determination. We also clarified that the post-hoc power analysis showed sufficient power to detect large effects, which is consistent with effect sizes reported in similar exercise intervention studies.

**Action Taken:** The Participants and Limitations sections have been revised.

Line numbers in the revised manuscript: Materials and Methods, Participants; Limitations.

**Comment (Dietary Factors):** The authors should also discuss the potential influence of dietary factors on the study outcomes. Since no information was provided regarding participants' dietary intake or possible changes in eating habits during the intervention, it is not possible to exclude the possibility that the observed improvements in anthropometric measures were partly attributable to dietary modifications rather than to the exercise programme alone. This potential confounding factor should be more thoroughly acknowledged and discussed.

**Our Response:** We agree with the reviewer. We have thoroughly expanded the discussion of this issue in both the Discussion and Limitations sections. We now explicitly state that participants may have consciously or unconsciously modified their dietary habits, which could have contributed to the observed changes. We also note that the absence of dietary monitoring precludes identifying any participants whose diet might have attenuated the expected improvements, making this a significant confounding factor.

**Action Taken:** The Discussion and Limitations sections have been revised.

Line numbers in the revised manuscript: Discussion, Paragraph 8; Limitations.

**Comment (Correlation Analysis):** The authors could also include a correlation analysis examining the relationship between changes in anthropometric measures and body image. Such an analysis would provide valuable insight into whether improvements in body image were associated with the observed anthropometric changes, thereby strengthening the interpretation of the findings.

**Our Response:** We thank the reviewer for this valuable suggestion. We have performed Spearman correlation analyses to examine the relationships between changes in body image scores and changes in anthropometric measurements. The results are now presented in the new Table 4 and discussed in the Discussion section. The strongest correlation was found between body image improvement and waist circumference reduction.

**Action Taken:** A new Table 4 and corresponding text have been added to the Results and Discussion sections.

Line numbers in the revised manuscript: Results, Correlation Between Body Image and Anthropometric Changes; Discussion.

**RESPONSES TO ACADEMIC EDITOR AND ADDITIONAL JOURNAL REQUIREMENTS**

**Editor's Note 1: Data Availability Statement**

**Comment:** You indicated that you will make your data available on acceptance. We strongly recommend all authors decide on a data sharing plan before acceptance.

**Our Response:** We have now prepared a fully anonymized dataset containing all raw data for the 40 participants. This dataset includes demographic characteristics, anthropometric measurements, and Body Image Scale scores. This file will be uploaded as a Supporting Information file (S1_Data.xlsx) with our revised submission.

**Editor's Note 2: Identifying Data in Supporting Information**

**Comment:** We note that there is identifying data in the Supporting Information file renamed_61fb3.xlsx. Please remove or anonymize all personal information and re-upload a fully anonymized data set.

**Our Response:** We have fully anonymized the dataset. The revised Supporting Information file contains only a participant ID number (1-40) and no personally identifiable information (no names, initials, specific dates, or any other identifiers). All data is in accordance with participant consent for public sharing.

**Editor's Note 3: Laboratory Protocols in protocols.io**

**Comment:** We recommend that you deposit your laboratory protocols in protocols.io to enhance the reproducibility of your results.

**Our Response:** We have heeded this suggestion and deposited a detailed, step-by-step protocol of our indoor cycling exercise intervention on protocols.io. To ensure reviewer anonymity, a blinded link has been provided in the Methods section of the manuscript: <https://www.protocols.io/blind/B1D0AE55878411F199810A58A9FEAC02>

**Editor's Note 4: ORCID Verification**

**Comment:** Please encourage your coauthors to verify their ORCID iD within the submission system before final acceptance.

**Our Response:** We thank the editor for this reminder. All coauthors have been contacted and have verified their ORCID iDs in the submission system.

We hope that these revisions have fully addressed all the concerns raised by the reviewers. We believe the manuscript is now significantly stronger and meets the publication criteria of PLOS ONE. We thank the editor and reviewers again for their time and valuable feedback.

We look forward to your positive decision.

Sincerely,

(Corresponding Author)
