# Supplementary material for: Effects of indoor cycling (spinning) exercise on body image and anthropometric measurements in women: An eight-week intervention study
Source: PLoS One. 2026 Oct 5;21(10):e0358959. doi: 10.1371/journal.pone.0358959 (PMC13637853; doi:10.1371/journal.pone.0358959)
Supplement: Peer review history

# Peer review: Decision Letter 0

**DOI:** 10.1371/journal.pone.0358959.r001

**Authors:** Raul Bartolomeu

24 Jul 2026

Dear Dr. Kasap,

Thank you for submitting your manuscript to PLOS ONE. After careful consideration, we feel that it has merit but does not fully meet PLOS ONE’s publication criteria as it currently stands. Therefore, we invite you to submit a revised version of the manuscript that addresses the points raised during the review process.

Please submit your revised manuscript by Sep 07 2026 11:59PM. If you will need more time than this to complete your revisions, please reply to this message or contact the journal office at plosone@plos.org. When you're ready to submit your revision, log on to https://www.editorialmanager.com/pone/ and select the 'Submissions Needing Revision' folder to locate your manuscript file.

- A letter that responds to each point raised by the academic editor and reviewer(s). You should upload this letter as a separate file labeled 'Response to Reviewers'.
- A marked-up copy of your manuscript that highlights changes made to the original version. You should upload this as a separate file labeled 'Revised Manuscript with Track Changes'.
- An unmarked version of your revised paper without tracked changes. You should upload this as a separate file labeled 'Manuscript'.

If you would like to make changes to your financial disclosure, please include your updated statement in your cover letter. Guidelines for resubmitting your figure files are available below the reviewer comments at the end of this letter.

If applicable, we recommend that you deposit your laboratory protocols in protocols.io to enhance the reproducibility of your results. Protocols.io assigns your protocol its own identifier (DOI) so that it can be cited independently in the future. For instructions see: https://journals.plos.org/plosone/s/submission-guidelines#loc-laboratory-protocols. Additionally, PLOS ONE offers an option for publishing peer-reviewed Lab Protocol articles, which describe protocols hosted on protocols.io. Read more information on sharing protocols at https://plos.org/protocols?utm_medium=editorial-email&utm_source=authorletters&utm_campaign=protocols.

As the corresponding author, your ORCID iD is verified in the submission system and will appear in the published article. PLOS supports the use of ORCID, and we encourage all coauthors to register for an ORCID iD and use it as well. Please encourage your coauthors to verify their ORCID iD within the submission system before final acceptance, as unverified ORCID iDs will not appear in the published article. *Only*  the individual author can complete the verification step; PLOS staff *cannot*  verify ORCID iDs on behalf of authors.

We look forward to receiving your revised manuscript.

Kind regards,

Raul Bartolomeu

Academic Editor

PLOS One

Journal requirements:

When submitting your revision, we need you to address these additional requirements.

1. Please ensure that your manuscript meets PLOS ONE's style requirements, including those for file naming. The PLOS ONE style templates can be found at

https://journals.plos.org/plosone/s/file?id=wjVg/PLOSOne_formatting_sample_main_body.pdf and

https://journals.plos.org/plosone/s/file?id=ba62/PLOSOne_formatting_sample_title_authors_affiliations.pdf

2. When completing the data availability statement of the submission form, you indicated that you will make your data available on acceptance. We strongly recommend all authors decide on a data sharing plan before acceptance, as the process can be lengthy and hold up publication timelines. Please note that, though access restrictions are acceptable now, your entire data will need to be made freely accessible if your manuscript is accepted for publication. This policy applies to all data except where public deposition would breach compliance with the protocol approved by your research ethics board. If you are unable to adhere to our open data policy, please kindly revise your statement to explain your reasoning and we will seek the editor's input on an exemption. Please be assured that, once you have provided your new statement, the assessment of your exemption will not hold up the peer review process.

3. We note that there is identifying data in the Supporting Information file <renamed_61fb3.xlsx>. Due to the inclusion of these potentially identifying data, we have removed this file from your file inventory. Prior to sharing human research participant data, authors should consult with an ethics committee to ensure data are shared in accordance with participant consent and all applicable local laws.

Data sharing should never compromise participant privacy. It is therefore not appropriate to publicly share personally identifiable data on human research participants. The following are examples of data that should not be shared:

-Name, initials, physical address

-Ages more specific than whole numbers

-Internet protocol (IP) address

-Specific dates (birth dates, death dates, examination dates, etc.)

-Contact information such as phone number or email address

-Location data

-ID numbers that seem specific (long numbers, include initials, titled “Hospital ID”) rather than random (small numbers in numerical order)

Data that are not directly identifying may also be inappropriate to share, as in combination they can become identifying. For example, data collected from a small group of participants, vulnerable populations, or private groups should not be shared if they involve indirect identifiers (such as sex, ethnicity, location, etc.) that may risk the identification of study participants.

Additional guidance on preparing raw data for publication can be found in our Data Policy (https://journals.plos.org/plosone/s/data-availability#loc-human-research-participant-data-and-other-sensitive-data) and in the following article: http://www.bmj.com/content/340/bmj.c181.long.

Please remove or anonymize all personal information, ensure that the data shared are in accordance with participant consent, and re-upload a fully anonymized data set. Please note that spreadsheet columns with personal information must be removed and not hidden as all hidden columns will appear in the published file.

If the reviewer comments include a recommendation to cite specific previously published works, please review and evaluate these publications to determine whether they are relevant and should be cited. There is no requirement to cite these works unless the editor has indicated otherwise.

[Note: HTML markup is below. Please do not edit.]

Reviewers' comments:

Reviewer's Responses to Questions

**Comments to the Author**

1. Is the manuscript technically sound, and do the data support the conclusions?

Reviewer #1: Yes

Reviewer #2: Partly

**********

2. Has the statistical analysis been performed appropriately and rigorously?

Reviewer #1: Yes

Reviewer #2: Yes

**********

3. Have the authors made all data underlying the findings in their manuscript fully available?

The PLOS Data policy

Reviewer #1: Yes

Reviewer #2: Yes

**********

4. Is the manuscript presented in an intelligible fashion and written in standard English?

Reviewer #1: Yes

Reviewer #2: Yes

**********

Reviewer #1: Abstract

The abstract clearly presents the main findings, but it omits essential information about the study design. In particular, it does not specify that this was a pre-post study without a control group, which affects the causal interpretation of the results (Shu et al., 2023, PLOS ONE, doi: 10.1371/journal.pone.0291045). Nor does it report the numerical values for the change in body image (137.30 to 159.00) or the absolute magnitude of the weight reduction (-3.82 kg). These data are presented in the tables and should be incorporated into the abstract to facilitate quantitative reading.

Introduction

Paragraph 2 (lines 61–71). Physical exercise is presented as a tool that may be considered for improving body image, but no distinction is made between aerobic and strength-based modalities. The meta-analysis by Fan et al. (2024, BMC Public Health, doi: 10.1186/s12889-024-20861-7) showed that aerobic exercise yields an effect size of g=0.63 for body image in women, compared with g=0.38 in combined programmes. This specificity should be mentioned to justify the choice of spinning.

Paragraph 4 (lines 78–84). The review by Chavarrias et al. (2019) is cited as general evidence, but it is omitted that most studies focused on indoor cycling have centred on women with obesity or overweight (Ratajczak et al., 2020, IJERPH, doi: 10.3390/ijerph17238718), which is relevant given that the sample in the present study (mean BMI 27.05 kg/m²) falls within the overweight range.

Materials and Methods

Participants (lines 97–115). No a priori sample size calculation is provided. Reporting post-hoc power (lines 170–175) does not replace an a priori calculation, and the authors themselves acknowledge its limitations. This should be clarified.

Study Design (lines 117–121). The pre-post design without a control group is the main methodological limitation and should be stated explicitly at this point, rather than only at the end of the manuscript.

Anthropometric Measurements (lines 152–158). Only one measurement per variable is reported. It would be advisable to specify whether duplicate or triplicate measurements were taken, as well as the intra-observer coefficient of variation, given that body image may additionally modulate beliefs about body shape (Gómez Chávez et al., 2022, RICCAFD, 11(2): 121-133, doi: 10.24310/riccafd.2022.v11i2.14684).

Results

Section 3 (Table 1, line 185). There is an inconsistency: the text (line 183) reports a mean BMI of 27.07 kg/m², whereas Table 1 gives 27.05 kg/m². This should be standardised.

Section 3 (Table 2, line 200). The change in shoulder circumference (107.78 to 107.90 cm) is virtually negligible and should be highlighted at the beginning of the section, rather than at the end, as an interpretative contrast that reflects the regional specificity of the aerobic stimulus.

Discussion

Paragraph 7 (lines 261–270). Despite the findings, the interpretation of the psychological effects of spinning is reasonable; however, it should be noted that in Latin American populations exercise does not always act as a moderating factor for psychological distress (Gómez Chávez et al., 2022, RICCAFD, 11(2): 121-133). This cultural variability should be addressed when discussing the generalisability of the findings.

Paragraph 8 (lines 271–279). The reduction in waist circumference (-4.5 cm) is described as “clinically significant”, but this is not contextualised in relation to dietary habits, which may mediate this response (Meza Miranda, 2024, RICCAFD, 13(3): 274-285, doi: https://doi.org/10.24310/riccafd.13.3.2024.19808).

Conclusion

Regarding the findings obtained, the conclusion is coherent, although, in my view, the assertion that spinning constitutes a “holistic approach” goes beyond what a design without a control group can support. The translational scope should therefore be qualified, and this term reserved for future studies using a randomised design, as the authors themselves acknowledge in lines 354–357.

Reviewer #2: The manuscript is well developed, addresses an interesting topic, and has the potential to be published. However, several issues should be clarified and addressed before it can be considered for publication:

- The authors should justify why a control group was not included, rather than simply stating that no control group was used and acknowledging this as a limitation.

- The authors should justify why an a priori sample size calculation was not performed. Although they acknowledge that the sample size is relatively small and that the findings cannot be generalized, this statement requires further justification. On what basis do the authors conclude that the sample is too small or not sufficiently representative? An a priori sample size calculation is essential to demonstrate that the study was adequately powered to detect the expected effects and to support the validity of the findings.

-The authors should also discuss the potential influence of dietary factors on the study outcomes. Since no information was provided regarding participants' dietary intake or possible changes in eating habits during the intervention, it is not possible to exclude the possibility that the observed improvements in anthropometric measures were partly attributable to dietary modifications rather than to the exercise programme alone. This potential confounding factor should be more thoroughly acknowledged and discussed.

-The authors could also include a correlation analysis examining the relationship between changes in anthropometric measures and body image. Such an analysis would provide valuable insight into whether improvements in body image were associated with the observed anthropometric changes, thereby strengthening the interpretation of the findings.

**********

what does this mean?). If published, this will include your full peer review and any attached files.

If you choose “no”, your identity will remain anonymous but your review may still be made public.

**Do you want your identity to be public for this peer review?**  For information about this choice, including consent withdrawal, please see our Privacy Policy

Reviewer #1: No

Reviewer #2: **Yes:**  Patrícia Filipa Saraiva de Sousa

**********

[NOTE: If reviewer comments were submitted as an attachment file, they will be attached to this email and accessible via the submission site. Please log into your account, locate the manuscript record, and check for the action link "View Attachments". If this link does not appear, there are no attachment files.]

To ensure your figures meet our technical requirements, please review our figure guidelines: https://journals.plos.org/plosone/s/figures

You may also use PLOS’s free figure tool, NAAS, to help you prepare publication quality figures: https://journals.plos.org/plosone/s/figures#loc-tools-for-figure-preparation.

NAAS will assess whether your figures meet our technical requirements by comparing each figure against our figure specifications.

# Author comment: Author response to Decision Letter 1

**DOI:** 10.1371/journal.pone.0358959.r002

28 Jul 2026

RESPONSES TO REVIEWER #1

Reviewer #1: Abstract

Comment: The abstract clearly presents the main findings, but it omits essential information about the study design. In particular, it does not specify that this was a pre-post study without a control group, which affects the causal interpretation of the results (Shu et al., 2023, PLOS ONE, doi: 10.1371/journal.pone.0291045). Nor does it report the numerical values for the change in body image (137.30 to 159.00) or the absolute magnitude of the weight reduction (-3.82 kg). These data are presented in the tables and should be incorporated into the abstract to facilitate quantitative reading.

Our Response: We thank the reviewer for this valuable suggestion. We have revised the abstract to explicitly state that this was a pre-test post-test intervention study without a control group and have added a cautionary note regarding causal interpretations. We have also incorporated the numerical values for the body image scores and the absolute weight reduction as requested.

Action Taken: The abstract has been revised accordingly.

Line numbers in the revised manuscript: Abstract section.

Reviewer #1: Introduction

Comment (Paragraph 2): Physical exercise is presented as a tool that may be considered for improving body image, but no distinction is made between aerobic and strength-based modalities. The meta-analysis by Fan et al. (2024, BMC Public Health, doi: 10.1186/s12889-024-20861-7) showed that aerobic exercise yields an effect size of g=0.63 for body image in women, compared with g=0.38 in combined programmes. This specificity should be mentioned to justify the choice of spinning.

Our Response: We agree with the reviewer and have added the comparison of effect sizes between aerobic (g=0.63) and combined (g=0.38) programs from the Zhang et al. [29] (Fan et al.) meta-analysis. This distinction now clearly justifies our choice of a purely aerobic intervention like spinning.

Action Taken: The Introduction section has been updated.

Line numbers in the revised manuscript: Introduction, Paragraph 2.

Comment (Paragraph 4): The review by Chavarrias et al. (2019) is cited as general evidence, but it is omitted that most studies focused on indoor cycling have centred on women with obesity or overweight (Ratajczak et al., 2020, IJERPH, doi: 10.3390/ijerph17238718), which is relevant given that the sample in the present study (mean BMI 27.05 kg/m²) falls within the overweight range.

Our Response: This is an excellent point. We have now explicitly noted that most studies on indoor cycling focus on overweight or obese women and highlighted that our sample (mean BMI 27.05 kg/m²) falls within this range, which strengthens the relevance of our work.

Action Taken: The Introduction section has been updated.

Line numbers in the revised manuscript: Introduction, Paragraph 4.

Reviewer #1: Materials and Methods

Comment (Participants): No a priori sample size calculation is provided. Reporting post-hoc power does not replace an a priori calculation, and the authors themselves acknowledge its limitations. This should be clarified.

Our Response: We acknowledge this limitation and have clarified the reasoning further. We have expanded the explanation in the Participants section to justify the use of a post-hoc power analysis and the exploratory nature of the study.

Action Taken: The Participants section has been revised.

Line numbers in the revised manuscript: Materials and Methods, Participants.

Comment (Study Design): The pre-post design without a control group is the main methodological limitation and should be stated explicitly at this point, rather than only at the end of the manuscript.

Our Response: We agree and have now explicitly stated the limitation of the single-group pre-post design in the Study Design section. We also provided a strong justification for this choice, emphasizing the exploratory aim of generating effect size estimates for future research.

Action Taken: The Study Design section has been significantly revised.

Line numbers in the revised manuscript: Materials and Methods, Study Design.

Comment (Anthropometric Measurements): Only one measurement per variable is reported. It would be advisable to specify whether duplicate or triplicate measurements were taken, as well as the intra-observer coefficient of variation, given that body image may additionally modulate beliefs about body shape.

Our Response: We thank the reviewer for this suggestion. We have now clarified in the Anthropometric Measurements section that all circumference measurements were taken twice, with a third measurement taken if the difference exceeded 0.5 cm. We have also reported the intra-observer coefficients of variation, which ranged from 0.5% to 1.2%, indicating acceptable reliability.

Action Taken: The Anthropometric Measurements section has been expanded.

Line numbers in the revised manuscript: Materials and Methods, Anthropometric Measurements.

Reviewer #1: Results

Comment (Section 3, Table 1): There is an inconsistency: the text reports a mean BMI of 27.07 kg/m², whereas Table 1 gives 27.05 kg/m². This should be standardised.

Our Response: We thank the reviewer for catching this error. The value has been standardised to 27.05 kg/m² in both the text and Table 1.

Action Taken: The text has been corrected.

Line numbers in the revised manuscript: Results, Demographic Characteristics of Participants.

Comment (Section 3, Table 2): The change in shoulder circumference (107.78 to 107.90 cm) is virtually negligible and should be highlighted at the beginning of the section, rather than at the end, as an interpretative contrast that reflects the regional specificity of the aerobic stimulus.

Our Response: We agree with the reviewer's interpretation. We have restructured the paragraph in the Results section to present the significant changes in body weight and other circumferences first, and then placed the non-significant shoulder circumference change at the end, emphasizing it as a contrast that highlights the regional specificity of the aerobic stimulus.

Action Taken: The Results section has been reorganized.

Line numbers in the revised manuscript: Results, Findings Related to Anthropometric Measurements.

Reviewer #1: Discussion

Comment (Paragraph 7): Despite the findings, the interpretation of the psychological effects of spinning is reasonable; however, it should be noted that in Latin American populations exercise does not always act as a moderating factor for psychological distress (Gómez Chávez et al., 2022, RICCAFD, 11(2): 121-133). This cultural variability should be addressed when discussing the generalisability of the findings.

Our Response: This is an important point. We have incorporated this perspective into the Discussion section, acknowledging that the psychological benefits of exercise may vary across cultural contexts, as demonstrated by Gómez Chávez et al. (2022). We now discuss the need for future research in diverse cultural settings to confirm the generalizability of our findings.

Action Taken: The Discussion section has been updated.

Line numbers in the revised manuscript: Discussion, Paragraph 7.

Comment (Paragraph 8): The reduction in waist circumference (-4.5 cm) is described as "clinically significant", but this is not contextualised in relation to dietary habits, which may mediate this response (Meza Miranda, 2024, RICCAFD, 13(3): 274-285, doi: https://doi.org/10.24310/riccafd.13.3.2024.19808).

Our Response: We fully agree and have significantly expanded our discussion of this point. We now explicitly acknowledge that the observed waist circumference reduction may be partially mediated by dietary factors, which we did not monitor. This is now discussed as a significant confounding factor in both the Discussion and Limitations sections.

Action Taken: The Discussion and Limitations sections have been revised.

Line numbers in the revised manuscript: Discussion, Paragraph 8; Limitations.

Reviewer #1: Conclusion

Comment: Regarding the findings obtained, the conclusion is coherent, although, in my view, the assertion that spinning constitutes a "holistic approach" goes beyond what a design without a control group can support. The translational scope should therefore be qualified, and this term reserved for future studies using a randomised design, as the authors themselves acknowledge in lines 354--357.

Our Response: We thank the reviewer for this important clarification. We have removed the term "holistic" and replaced it with more cautious wording, such as "a promising exercise approach." We have also strengthened the concluding statements to emphasize that these findings are preliminary and require confirmation through future randomized controlled trials.

Action Taken: The Conclusion section has been revised.

Line numbers in the revised manuscript: Conclusion.

RESPONSES TO REVIEWER #2

Reviewer #2: General Comment

Comment: The manuscript is well developed, addresses an interesting topic, and has the potential to be published. However, several issues should be clarified and addressed before it can be considered for publication.

Our Response: We thank the reviewer for their positive and constructive feedback. We have carefully addressed each of the points raised, as detailed below.

Comment (Control Group): The authors should justify why a control group was not included, rather than simply stating that no control group was used and acknowledging this as a limitation.

Our Response: We have provided a detailed justification in the Study Design section. We now explain that the absence of a control group was due to practical constraints in participant recruitment and adherence, as well as the exploratory nature of this investigation aimed at generating effect size estimates. We also emphasize that the primary objective was to assess within-subject changes to inform future randomized controlled trials, not to establish causal inference.

Action Taken: The Study Design section has been significantly revised.

Line numbers in the revised manuscript: Materials and Methods, Study Design.

Comment (Sample Size): The authors should justify why an a priori sample size calculation was not performed. Although they acknowledge that the sample size is relatively small and that the findings cannot be generalized, this statement requires further justification. On what basis do the authors conclude that the sample is too small or not sufficiently representative? An a priori sample size calculation is essential to demonstrate that the study was adequately powered to detect the expected effects and to support the validity of the findings.

Our Response: We have significantly expanded our explanation in both the Participants and Limitations sections. The lack of an a priori calculation is justified by the absence of comparable effect size estimates in this specific population and intervention context at the time of study planning. The exploratory nature of the study justified a flexible approach to sample size determination. We also clarified that the post-hoc power analysis showed sufficient power to detect large effects, which is consistent with effect sizes reported in similar exercise intervention studies.

Action Taken: The Participants and Limitations sections have been revised.

Line numbers in the revised manuscript: Materials and Methods, Participants; Limitations.

Comment (Dietary Factors): The authors should also discuss the potential influence of dietary factors on the study outcomes. Since no information was provided regarding participants' dietary intake or possible changes in eating habits during the intervention, it is not possible to exclude the possibility that the observed improvements in anthropometric measures were partly attributable to dietary modifications rather than to the exercise programme alone. This potential confounding factor should be more thoroughly acknowledged and discussed.

Our Response: We agree with the reviewer. We have thoroughly expanded the discussion of this issue in both the Discussion and Limitations sections. We now explicitly state that participants may have consciously or unconsciously modified their dietary habits, which could have contributed to the observed changes. We also note that the absence of dietary monitoring precludes identifying any participants whose diet might have attenuated the expected improvements, making this a significant confounding factor.

Action Taken: The Discussion and Limitations sections have been revised.

Line numbers in the revised manuscript: Discussion, Paragraph 8; Limitations.

Comment (Correlation Analysis): The authors could also include a correlation analysis examining the relationship between changes in anthropometric measures and body image. Such an analysis would provide valuable insight into whether improvements in body image were associated with the observed anthropometric changes, thereby strengthening the interpretation of the findings.

Our Response: We thank the reviewer for this valuable suggestion. We have performed Spearman correlation analyses to examine the relationships between changes in body image scores and changes in anthropometric measurements. The results are now presented in the new Table 4 and discussed in the Discussion section. The strongest correlation was found between body image improvement and waist circumference reduction.

Action Taken: A new Table 4 and corresponding text have been added to the Results and Discussion sections.

Line numbers in the revised manuscript: Results, Correlation Between Body Image and Anthropometric Changes; Discussion.

RESPONSES TO ACADEMIC EDITOR AND ADDITIONAL JOURNAL REQUIREMENTS

Editor's Note 1: Data Availability Statement

Comment: You indicated that you will make your data available on acceptance. We strongly recommend all authors decide on a data sharing plan before acceptance.

Our Response: We have now prepared a fully anonymized dataset containing all raw data for the 40 participants. This dataset includes demographic characteristics, anthropometric measurements, and Body Image Scale scores. This file will be uploaded as a Supporting Information file (S1_Data.xlsx) with our revised submission.

Editor's Note 2: Identifying Data in Supporting Information

Comment: We note that there is identifying data in the Supporting Information file renamed_61fb3.xlsx. Please remove or anonymize all personal information and re-upload a fully anonymized data set.

Our Response: We have fully anonymized the dataset. The revised Supporting Information file contains only a participant ID number (1-40) and no personally identifiable information (no names, initials, specific dates, or any other identifiers). All data is in accordance with participant consent for public sharing.

Editor's Note 3: Laboratory Protocols in protocols.io

Comment: We recommend that you deposit your laboratory protocols in protocols.io to enhance the reproducibility of your results.

Our Response: We have heeded this suggestion and deposited a detailed, step-by-step protocol of our indoor cycling exercise intervention on protocols.io. To ensure reviewer anonymity, a blinded link has been provided in the Methods section of the manuscript: https://www.protocols.io/blind/B1D0AE55878411F199810A58A9FEAC02

Editor's Note 4: ORCID Verification

Comment: Please encourage your coauthors to verify their ORCID iD within the submission system before final acceptance.

Our Response: We thank the editor for this reminder. All coauthors have been contacted and have verified their ORCID iDs in the submission system.

We hope that these revisions have fully addressed all the concerns raised by the reviewers. We believe the manuscript is now significantly stronger and meets the publication criteria of PLOS ONE. We thank the editor and reviewers again for their time and valuable feedback.

We look forward to your positive decision.

Sincerely,

(Corresponding Author)

Attachment Submitted filename: Response to Reviewers.docx

# Peer review: Decision Letter 1

**DOI:** 10.1371/journal.pone.0358959.r003

**Authors:** Raul Bartolomeu

9 Sep 2026

Effects of Indoor Cycling (Spinning) Exercise on Body Image and Anthropometric Measurements in Women: An Eight-Week Intervention Study

PONE-D-26-08250R1

Dear Dr. Kasap,

We’re pleased to inform you that your manuscript has been judged scientifically suitable for publication and will be formally accepted for publication once it meets all outstanding technical requirements.

Within one week, you’ll receive an e-mail detailing the required amendments. When these have been addressed, you’ll receive a formal acceptance letter and your manuscript will be scheduled for publication.

An invoice will be generated when your article is formally accepted. Please note, if your institution has a publishing partnership with PLOS and your article meets the relevant criteria, all or part of your publication costs will be covered. Please make sure your user information is up-to-date by logging into Editorial Manager at Editorial Manager® and clicking the ‘Update My Information' link at the top of the page. For questions related to billing, please contact billing support.

If your institution or institutions have a press office, please notify them about your upcoming paper to help maximize its impact. If they’ll be preparing press materials, please inform our press team as soon as possible -- no later than 48 hours after receiving the formal acceptance. Your manuscript will remain under strict press embargo until 2 pm Eastern Time on the date of publication. For more information, please contact onepress@plos.org.

Kind regards,

Raul Bartolomeu

Academic Editor

PLOS One

Additional Editor Comments (optional):

Reviewers' comments:

Reviewer's Responses to Questions

**Comments to the Author**

Reviewer #2: All comments have been addressed

**********

2. Is the manuscript technically sound, and do the data support the conclusions?

Reviewer #2: Yes

**********

3. Has the statistical analysis been performed appropriately and rigorously?

Reviewer #2: Yes

**********

4. Have the authors made all data underlying the findings in their manuscript fully available?

The PLOS Data policy

Reviewer #2: Yes

**********

5. Is the manuscript presented in an intelligible fashion and written in standard English?

Reviewer #2: Yes

**********

Reviewer #2: (No Response)

**********

what does this mean?). If published, this will include your full peer review and any attached files.

If you choose “no”, your identity will remain anonymous but your review may still be made public.

**Do you want your identity to be public for this peer review?**  For information about this choice, including consent withdrawal, please see our Privacy Policy

Reviewer #2: **Yes:**  Patrícia Sousa

**********

# Editor report: Acceptance letter

**DOI:** 10.1371/journal.pone.0358959.r004

**Authors:** Raul Bartolomeu

PONE-D-26-08250R1

PLOS One

Dear Dr. Kasap,

I'm pleased to inform you that your manuscript has been deemed suitable for publication in PLOS One. Congratulations! Your manuscript is now being handed over to our production team.

At this stage, our production department will prepare your paper for publication. This includes ensuring the following:

* All references, tables, and figures are properly cited

* All relevant supporting information is included in the manuscript submission,

* There are no issues that prevent the paper from being properly typeset

You will receive further instructions from the production team, including instructions on how to review your proof when it is ready. Please keep in mind that we are working through a large volume of accepted articles, so please give us a few days to review your paper and let you know the next and final steps.

Lastly, if your institution or institutions have a press office, please let them know about your upcoming paper now to help maximize its impact. If they'll be preparing press materials, please inform our press team within the next 48 hours. Your manuscript will remain under strict press embargo until 2 pm Eastern Time on the date of publication. For more information, please contact onepress@plos.org.

You will receive an invoice from PLOS for your publication fee after your manuscript has reached the completed accept phase. If you receive an email requesting payment before acceptance or for any other service, this may be a phishing scheme. Learn how to identify phishing emails and protect your accounts at https://explore.plos.org/phishing.

If we can help with anything else, please email us at customercare@plos.org.

Thank you for submitting your work to PLOS One and supporting open access.

Kind regards,

PLOS One Editorial Office Staff

on behalf of

Dr. Raul Bartolomeu

Academic Editor

PLOS One
